# Supplementary material for: Optimizing respiratory virus surveillance networks using uncertainty propagation
Source: Nat Commun. 2021 Jan 11;12:222. doi: 10.1038/s41467-020-20399-3 (PMC7801666; doi:10.1038/s41467-020-20399-3)
Supplement: Supplementary file 1 — Supplementary Information [file 41467_2020_20399_MOESM1_ESM.pdf]

Supplementary Information for
Optimizing respiratory virus surveillance networks using uncertainty propagation

Sen Pei[1*], Xian Teng[2], Paul Lewis[3], Jeffrey Shaman[1*]

[1]Department of Environmental Health Sciences, Mailman School of Public Health, Columbia University, New York, NY 10032, USA
[2]School of Computing and Information, University of Pittsburgh, Pittsburgh, PA 15260, USA
[3]Integrated Biosurveillance Section, Armed Forces Health Surveillance Branch, Silver Spring, MD 20904, USA

*Corresponding authors. E-mail: sp3449@cumc.columbia.edu (SP), jls106@cumc.columbia.edu (JS).

Note 1 Data description

We used patient syndromic and laboratory test data from the US Department of Defense (DoD) Armed Forces Health Surveillance Branch (AFHSB) to estimate state-level influenza activity. The syndromic data contain patient line records from 81,461,411 influenza-like illness (ILI) related visits to permanent military treatment facilities (MTFs), each with a diagnosis containing at least one of the 29 International Classification of Diseases (ICD-9) codes associated with ILI (*1*). These records span a period of nearly 18 years, from January 1st, 2000 to November 28th, 2017, and include approximately 1,000 MTFs located in 42 states in the United States. Patients include both military personnel and other beneficiaries (e.g., spouses and children). We define *ILI rate* as the probability a patient seeking medical care is diagnosed with ILI symptoms (i.e., cough and/or sore throat and a fever above 100°F). To estimate the ILI rate among all medical-seeking visits, we normalize the weekly ILI-related visits by the weekly total number of visits for any reason to MTFs within each state.

Because ILI is not exclusively specified for influenza, samples from a small portion of patients with ILI symptoms were sent out for further laboratory testing to confirm which are positive for influenza type A and type B. The laboratory data contain the test results from 845,081 samples collected during October 1st, 2006 to January 8th, 2018. Among all confirmed cases, influenza type A accounts for a larger proportion of infections (type A: 100,443 positive cases; type B: 25,937 positive cases); therefore we only focused on influenza type A in this study. We define *positivity rate* as the probability an ILI sample tested positive for influenza type A, estimated by the fraction of weekly influenza type A positive cases among all tested samples within each state. The weekly *ILI+ rate* in each state, defined as the probability a person seeking medical care is infected with influenza type A, is calculated as the product of the weekly ILI rate and the concurrent weekly influenza type A positivity rate. Unlike ILI, the ILI+ rate provides a

clearer signal of influenza type A activity among people visiting MTFs (*2*). To ensure sufficient surveillance sample size, in our analysis and retrospective forecasting, we omitted the 7 states with less than 70,000 total ILI-related visits. Further, we restricted retrospective forecasting to the nine consecutive flu seasons from 2008-2009 to 2016-2017, as laboratory testing before 2008 was less frequent. The AFHSB data, though only covering a small fraction of the total US population, have been found to agree well with the surveillance data reported by US Centers of Disease Control and Prevention (CDC) during the 2009 H1N1 pandemic season (*3*). The estimated weekly *ILI rates*, *positivity rates* and *ILI+ rates* for 35 states during 9 seasons are shown in Fig. 1A-C. Sample numbers are reported in Fig. 1D-F.

Local absolute humidity (AH) conditions for each state and county were obtained from North American Land Data Assimilation System data (*4*). A daily AH climatology of conditions averaged over a 24-year period from 1979 to 2002 was used. County-to-county commuting data, obtained from the 2009-2013 American Community Surveys, were used to approximate human movement. This dataset, publicly available from the United States Census Bureau website (*5*), provides commuting population estimates across all US counties. Given that the survey period (2009-2013) is within the forecast seasons (2008-2017), we assume the commuting patterns reported in the census survey data are representative of the study period. For state-level retrospective forecasting, we transformed the county-level commuting data to state level by only considering cross-state commuting.

HMPV and CoV infection rates in the selected 35 US states are estimated as the weekly ILI rate multiplied with concurrent weekly laboratory test positivity rates, termed HMPV+ and CoV+. From the beginning of 2013 to the end of 2017, 44,887 and 27,435 tests were performed for HMPV and CoV, respectively. In retrospective forecasts, we used 4 seasons of HMPV+ and CoV+ data from 2013-2014 to 2016-2017.

Note 2 The networked forecasting system

The model-data assimilation framework

Originally developed for numerical weather prediction, the model-data assimilation (M/D/A) framework is now increasingly used for real-time forecasting of infectious disease. In an M/D/A forecast system, a dynamical model describing the transmission process of an infectious disease is coupled with a data assimilation algorithm, which is used in conjunction with observations to iteratively update model variables and parameters (*6*). The optimized model is then integrated into the future to generate real-time predictions. Specifically, at each observation time, two procedures are performed: 1) *data assimilation*, which updates the model state using observations, and 2) *model integration*, which evolves the system to the next observation time. In operational forecasting practice, an ensemble of model trajectories is maintained to represent the distribution of system states. As a result, the M/D/A system generates probabilistic forecasts. To date, this system has been successfully applied to the inference and forecast of a range of infectious diseases, including influenza (*7–15*), dengue (*16*), RSV (*17*), West Nile virus (*18*), Ebola (*19*), and antibiotic-resistant pathogens (*20*).

The metapopulation model

We use a metapopulation model to simulate the spatiotemporal transmission of influenza across locations (*10*). The population in each location is classified into three compartments: susceptible (S), infected (I), recovered (R). Within each location, we assume individuals are well mixed and that transmission dynamics follow a typical susceptible-infected-recovered-susceptible (SIRS) model. Denote the total, susceptible and infected population in location i as N_i, S_i and I_i, respectively. A susceptible individual becomes infected with probability $\beta_i(t)$ upon contact with an infected person; an infected person recovers with probability $1/D$; and a recovered individual loses immunity with probability $1/L$. Here, D is the average du-

ration of infection, and L is the average duration of immunity. In particular, for influenza, the transmission rate, $\beta_i(t)$, is modulated by local AH conditions through $R_0^i(t) = \beta_i(t)D = \exp(a \times q_i(t) + b) + R_{0min}$ (*21, 22*), where $R_0^i(t)$ is the basic reproductive number at location i, and $q_i(t)$ is daily specific humidity, a measure of AH. The parameter a is estimated from laboratory experiments of the impact of AH on influenza virus survival, $a = -180$ and $b = \log(R_{0max} - R_{0min})$ (R_{0max} and R_{0min} are parameters representing the maximum and minimum daily basic reproductive numbers). For HMPV and CoV, we assume the transmission rate $\beta_i(t)$ is constant and same across locations.

In the metapopulation model, different locations are connected by human mobility (*23–26*). In particular, we assume a fraction of people randomly circulate among all locations following a Markov process. Previous theoretical analysis demonstrated that such diffusive random movement plays a major role in transporting infections (*27*). In practice, however, detailed information describing human movement is not available in real time other than the coarse-grained work commuting data reported in census surveys. To address this problem, we assume the volume of random movement between two locations is proportional to the average number of commuters between them (*10*). Denote C_j^i as the number of commuters living in location i and commuting to work in location j. The number of visitors from location i to j is assumed to be $\theta\bar{C}_j^i \equiv \theta(C_j^i + C_i^j)/2$, where θ is an adjustable parameter. The evolutions of I_i and S_i are described by the following transmission equations:

$$\frac{dI_i}{dt} = \frac{\beta_i(t)S_iI_i}{N_i} - \frac{I_i}{D} - \frac{\theta I_i}{N_i}\sum_{j\neq i}\bar{C}_j^i + \theta\sum_{j\neq i}\bar{C}_i^j\frac{I_j}{N_j}, \quad (1)$$

$$\frac{dS_i}{dt} = \frac{N_i - S_i - I_i}{L} - \frac{\beta_i(t)S_iI_i}{N_i} - \frac{\theta S_i}{N_i}\sum_{j\neq i}\bar{C}_j^i + \theta\sum_{j\neq i}\bar{C}_i^j\frac{S_j}{N_j}. \quad (2)$$

The latter two terms on the right hand side of Equations 1 and 2 represent the outgoing and incoming population. For instance, $\theta I_i \sum_{j\neq i}\bar{C}_j^i/N_i$ is the number of infected individuals leaving location i, and $\theta\sum_{j\neq i}\bar{C}_i^j I_j/N_j$ is the number of infections introduced from other locations.

Models with a similar framework have been widely used in simulating and forecasting infectious disease spread (*10, 28–33*).

Data assimilation

In real-time forecasting, not all variables and parameters in the metapopulation model are observed. For influenza, surveillance systems typically report only weekly incidence or ILI+ rates (as in this study). Important model variables (e.g., S_i and I_i) and parameters (e.g., R_{0max}, R_{0min}, L, D and θ) remain unknown. To estimate the unobserved variables and parameters from limited observations, a number of data assimilation techniques have been developed (*34–38*).

Denote the observation vector and state vector at time t as $\mathbf{y}_t^o$ and $\mathbf{z}_t$, respectively. In particular, the observation vector for a system with m locations is defined as $\mathbf{y}_t^o = (y_1^o, \cdots, y_m^o)^T$, where y_i^o is the observed ILI+ rate in location i. The state vector is defined as

$$\mathbf{z}_t = (I_1, S_1, y_1, \cdots, I_m, S_m, y_m, R_{0max}, R_{0min}, L, D, \theta)^T,$$

including unobserved state variables (infected population I_i and susceptible population S_i in location i), observed state variables (y_i, the ILI+ rate in location i, which is calculated during model integration as the new incidence rate and scaled to ILI+ rate) and parameters (R_{0max}, R_{0min}, L, D and θ). Note that we omit the time indicator t in y_i^o, I_i, S_i and y_i within $\mathbf{y}_t^o$ and $\mathbf{z}_t$ for notational convenience. In data assimilation, the prior distribution of the system state at time t, $p(\mathbf{z}_t|\mathbf{y}_{1:t-1}^o)$, is updated to the posterior distribution $p(\mathbf{z}_t|\mathbf{y}_{1:t}^o)$ using Bayes rule: $p(\mathbf{z}_t|\mathbf{y}_{1:t}^o) \propto p(\mathbf{z}_t|\mathbf{y}_{1:t-1}^o)p(\mathbf{y}_t^o|\mathbf{z}_t)$, where $p(\mathbf{y}_t^o|\mathbf{z}_t)$ is the likelihood of observing $\mathbf{y}_t^o$ given the prior state $\mathbf{z}_t$.

Different techniques for implementing this update result in different data assimilation algorithms. In this study, we employ the Ensemble Adjustment Kalman Filter (EAKF) (*34*), a computationally efficient data assimilation algorithm commonly used in numerical weather prediction and suited for application to high-dimensional systems such as the metapopulation model

employed here. In particular, unlike particle filter (or Sequential Monte Carlo) approaches, the EAKF can estimate high-dimensional systems using a relatively small ensemble size. The EAKF assumes a Gaussian distribution for both the prior and likelihood, allowing the parameterization of the distribution of the system state using only the first two moments – mean and covariance. The posterior mean and covariance are thus obtained through the convolution of two Gaussian distributions. In implementation, the distribution of the system state is represented by an ensemble of state vectors. During data assimilation, the ensemble members are updated deterministically so that the ensemble mean and covariance of the observed state variables in the posterior match their theoretical values, while higher moments of the prior are preserved. For unobserved state variables and parameters, their adjustments in the EAKF are linearly related to the adjustments of the observed state variables through their covariance, which can be calculated directly from the ensemble.

In data assimilation, we use observations $\mathbf{y}_t^o$ obtained from multiple locations to update the system state $\mathbf{z}_t$. Specifically, we loop through all available observations y_i^o sequentially to adjust each variable/parameter z in the state vector. Here we drop the location indicator i and denote each observation as y^o. For each ensemble member k, the observed variable y corresponding to y^o is updated through

$$y_{post}^{(k)} = \frac{\sigma_{y^o}^2}{\sigma_{y^o}^2 + \sigma_{y,prior}^2}\bar{y}_{prior} + \frac{\sigma_{y,prior}^2}{\sigma_{y^o}^2 + \sigma_{y,prior}^2}y^o + \sqrt{\frac{\sigma_{y^o}^2}{\sigma_{y^o}^2 + \sigma_{y,prior}^2}}(y_{prior}^{(k)} - \bar{y}_{prior}), \tag{3}$$

where $y_{prior}^{(k)}$ and $y_{post}^{(k)}$ are the prior and posterior of the observed state variable y for the kth ensemble member, $\bar{y}_{prior}$ is the prior mean of y, $\sigma_{y,prior}^2$ is the prior variance of y, and $\sigma_{y^o}^2$ is the variance of the observation y^o. For other variables/parameters z, the posterior is obtained through

$$z_{post}^{(k)} = z_{prior}^{(k)} + \frac{cov(\{z_{prior}\}, \{y_{prior}\})}{\sigma_{z,prior}^2}(y_{post}^{(k)} - y_{prior}^{(k)}), \tag{4}$$

where $z_{prior}^{(k)}$ and $z_{post}^{(k)}$ are the prior and posterior of the unobserved state variable z in the kth

ensemble member, $\sigma^2_{z,prior}$ is the prior variance of z, and $cov(\{z_{prior}\}, \{y_{prior}\})$ is the covariance between the prior of z and the prior of y, calculated from the ensembles $\{z_{prior}\}$ and $\{y_{prior}\}$. We note that the EAKF can be implemented even when some locations have no surveillance data, as shown in Fig. 1A.

Retrospective forecasting with incomplete surveillance data

In a networked forecasting system, data streams from multiple locations can be employed collectively to generate forecasts. That is, observations from one location can be used to adjust the model state in other locations. It is thus sensible to ask how well a networked forecasting system performs with only a partial complement of surveillance data. Addressing this question is the first step towards developing cost-effective surveillance and forecasting systems in circumstances where resources are constrained. Here we performed a state-level experiment comparing the accuracy of forecasts obtained from complete and partial surveillance data. Specifically, for each focal state i, we performed: 1) retrospective forecasting with data from all 35 state locations, including location i; and 2) retrospective forecasting with data from all state locations except location i. We then inspected the change of forecast mean absolute error (MAE) for predictions of incidence 1 to 4 weeks in the future caused by the omission of surveillance data from one state location. Figure 1C and Figure 2 show the results averaged over these 35 experiments. The forecasts were not substantially degraded by the omission of data from a single surveillance site. This preliminary result motivated further exploration of the heterogeneous importance of data sources from different locations, and the design of optimal surveillance networks for influenza surveillance and forecast.

Note 3 Uncertainty propagation and forecast accuracy

The objective of infectious disease surveillance is to use noisy incidence data to infer the current disease situation. Here, in the networked forecasting system, we use observed imperfect ILI+

rates to estimate the model state, and project the model from the estimated state into the future to generate forecasts. Due to observational error, the estimated model state (i.e., the posterior distribution) has uncertainty around the mean estimate (i.e., the posterior mean). Such uncertainty, measured by the standard deviation of the posterior distribution, reflects our confidence in the mean estimate.

In principle, if the dynamical model reliably describes the influenza transmission process, the forecast accuracy is determined by 1) the bias of the mean estimate from the true model state, and 2) the uncertainty around the mean estimate. Ideally, the surveillance network should be designed to generate unbiased estimation with a low level of uncertainty. Unfortunately, in reality, the true system state (e.g., the true infected population, basic reproductive number, etc.) is unknown. As a consequence, it is difficult to measure the discrepancy from the mean estimate to the true system state, which we here refer to as *estimation bias*. Although the actual estimation bias is unknown, as long as the mean model estimate does not diverge from the truth (i.e., the truth is captured by the posterior), it is reasonable to assume that the estimation bias is smaller than or of the same order of magnitude as the standard deviation of the posterior, as illustrated in the Fig. 3. In particular, the estimation bias can be regarded as a perturbation on the true model state and undergoes a similar spatiotemporal propagation of error as the uncertainty (i.e., standard deviation) does during model integration (*39*). As a consequence, the growth of estimation bias can be partially and indirectly reflected by the propagation of uncertainty. In our recent work, we found that the growth of error in the model state plays a major role in degrading forecast accuracy (*39*).

Following the above reasoning, we conclude that, for a well-behaved forecasting system without divergence, suppressing uncertainty propagation, i.e. the dynamic growth of error during model integration, would in effect suppress the growth of estimation bias as well. In addition, reduced uncertainty propagation would lead to more precise predictions with lower

variance among individual forecasts, indicating greater certainty. The relationship between uncertainty and forecast accuracy has been corroborated in real-time and retrospective forecasting of influenza (*6–8,10*). In these works, predictions with a lower uncertainty generally have higher accuracy. Based on this line of reasoning, we therefore propose to identify optimal influenza surveillance network configurations by optimizing the suppression of uncertainty propagation.

Note 4 Quantification of observation error variance

For each observation y^o, running the EAKF algorithm in Eq. (3) requires the input of the observation error variance (OEV), $\sigma^2_{y^o}$. However, the actual variance of y^o, $\sigma^2_{y^o}$, is unknown as only one data point is observed per site each week. Further, the quality of the surveillance data collected from different locations is quite heterogeneous and depends on the number of samples taken at each location. To represent this heterogeneity, we quantify $\sigma^2_{y^o}$ in different locations using sample information from the AFHSB dataset.

Assume that the local ILI rate and positivity rate at time t are represented by two random variables ili_t and pr_t, respectively. Recall that ili_t is the probability that a patient who visits a doctor is diagnosed with ILI symptoms, and pr_t is the probability that an ILI sample tests positive for influenza type A. The observed ILI rate and positivity rate, denoted by $\overline{ili}_t$ and $\overline{pr}_t$, are sample means calculated from n_t^{visit} medical visits and n_t^{test} laboratory tests. Precisely, the variance of the *sample mean* is related to sample size through

$$\sigma^2_{\overline{ili}_t} = \frac{\sigma^2_{ili_t}}{n_t^{visit}} \tag{5}$$

and

$$\sigma^2_{\overline{pr}_t} = \frac{\sigma^2_{pr_t}}{n_t^{test}}, \tag{6}$$

where $\sigma^2_{\overline{ili}_t}$ and $\sigma^2_{\overline{pr}_t}$ are the variances of sample means $\overline{ili}_t$ and $\overline{pr}_t$; $\sigma^2_{ili_t}$ and $\sigma^2_{pr_t}$ are the variances of the random variables ili_t and pr_t.

The observed ILI+ rate at time t is the product of the two sample means $\overline{ili}_t \times \overline{pr}_t$. Its variance is determined by

$$\sigma^2_{\overline{ili}_t \times \overline{pr}_t} = \sigma_{\overline{ili}_t^2, \overline{pr}_t^2} + (\sigma^2_{\overline{ili}_t} + \mu^2_{\overline{ili}_t})(\sigma^2_{\overline{pr}_t} + \mu^2_{\overline{pr}_t}) - (\sigma_{\overline{ili}_t, \overline{pr}_t} + \mu_{\overline{ili}_t}\mu_{\overline{pr}_t})^2, \tag{7}$$

where $\sigma_{\overline{ili}_t^2, \overline{pr}_t^2}$ is the covariance between $\overline{ili}_t^2$ and $\overline{pr}_t^2$; $\sigma_{\overline{ili}_t, \overline{pr}_t}$ is the covariance between $\overline{ili}_t$ and $\overline{pr}_t$; and $\mu_{\overline{ili}_t}$ and $\mu_{\overline{pr}_t}$ are the expectances of $\overline{ili}_t$ and $\overline{pr}_t$. Note that $\mu_{\overline{ili}_t} = \mu_{ili_t}$ and $\mu_{\overline{pr}_t} = \mu_{pr_t}$.

As the observed ILI rate $\overline{ili}_t$ and positivity rate $\overline{pr}_t$ are correlated, we need to estimate the terms $\sigma_{\overline{ili}_t^2, \overline{pr}_t^2}$ and $\sigma_{\overline{ili}_t, \overline{pr}_t}$. Denote r_1 (r_2) as the Pearson correlation coefficient between $\overline{ili}_t^2$ and $\overline{pr}_t^2$ ($\overline{ili}_t$ and $\overline{pr}_t$). This yields

$$\sigma_{\overline{ili}_t^2, \overline{pr}_t^2} = r_1 \sigma_{\overline{ili}_t^2} \sigma_{\overline{pr}_t^2} \tag{8}$$

and

$$\sigma_{\overline{ili}_t, \overline{pr}_t} = r_2 \sigma_{\overline{ili}_t} \sigma_{\overline{pr}_t}. \tag{9}$$

In practice, r_1 and r_2 can be computed from the observed time series for $\overline{ili}_t$ and $\overline{pr}_t$. Additionally, if ili_t and pr_t are Gaussian distributed: $ili_t \sim \mathcal{N}(\mu_{ili_t}, \sigma^2_{ili_t})$ and $pr_t \sim \mathcal{N}(\mu_{pr_t}, \sigma^2_{pr_t})$, we have (*40*)

$$\sigma_{ili_t^2} \approx \sqrt{2}\mu_{ili_t}\sigma_{ili_t} \tag{10}$$

and

$$\sigma_{pr_t^2} \approx \sqrt{2}\mu_{pr_t}\sigma_{pr_t}. \tag{11}$$

Using Eqs. (5)-(11), we expand Eq. (7) to

$$\sigma^2_{\overline{ili}_t \times \overline{pr}_t} = \frac{2(r_1 - r_2)}{\sqrt{n_t^{visit} n_t^{test}}} \mu_{ili_t}\mu_{pr_t}\sigma_{ili_t}\sigma_{pr_t} + \frac{1 - r_2^2}{n_t^{visit} n_t^{test}} \sigma^2_{ili_t}\sigma^2_{pr_t} + \frac{\mu^2_{ili_t}\sigma^2_{pr_t}}{n^{test_t}} + \frac{\mu^2_{pr_t}\sigma^2_{ili_t}}{n_t^{visit}}. \tag{12}$$

In the data, we observe that $n_t^{test}/n_t^{visit} \sim O(10^{-3})$ (Fig. 1F) such that the last term of Eq. 12 can be neglected. If the standard deviations of ili_t and pr_t are of the same order as their mean values, i.e. $\sigma_{ili_t} \sim O(\mu_{ili_t})$ and $\sigma_{pr_t} \sim O(\mu_{pr_t})$, the first 2 terms of Eq. (12) can also

be neglected. We thus finally derive the approximate analytical form for observational error variance, $\sigma^2_{y^o}$:

$$\sigma^2_{y^o} = \sigma^2_{\overline{ili}_t \times \overline{pr}_t} \approx \frac{\mu^2_{ili_t}\sigma^2_{pr_t}}{n^{test}_t}. \tag{13}$$

Equation (13) reveals that $\sigma^2_{y^o}$ is determined by the mean value of the ILI rate, μ_{ili_t}, the variance of the positivity rate, $\sigma^2_{pr_t}$, and the number of laboratory tests, n^{test}_t. In particular, given the large number of medical visits $n^{visit}_t \sim O(10^3 - 10^5)$, μ_{ili_t} can be approximated by the observed ILI rate $\overline{ili}_t$. The number of laboratory tests n^{test}_t is available from the data. However, the exact form of $\sigma^2_{pr_t}$ is unknown. To solve this problem, here we define a heuristic estimate of $\sigma^2_{pr_t}$ per (*6–8, 10*):

$$\sigma^2_{pr_t} = \sigma^2_0 + \nu \left(\sum_{t'=t-2}^{t} \overline{pr}_{t'}/3 \right)^2. \tag{14}$$

Note that $\sigma^2_{pr_t}$ is a baseline variance σ^2_0 plus a term proportional to the square of the average observed positivity rate during week $t-2$ to week t. In summary, the form of $\sigma^2_{y^o}$ (the observational error variance) is:

$$\sigma^2_{y^o} = \frac{\overline{ili}^2_t}{n^{test}_t} \left[\sigma^2_0 + \nu \left(\sum_{t'=t-2}^{t} \overline{pr}_{t'}/3 \right)^2 \right]. \tag{15}$$

To find suitable values of σ^2_0 and ν, we performed retrospective forecasting in the 35 state locations across 9 seasons using different pairs of $(\sigma_0, \sqrt{\nu})$, and selected the values producing the most accurate forecasts. Specifically, in each season, we generated 30 weekly forecasts of ILI+ for 1- to 4-weeks ahead (denoted as X1 to X4). In particular, we did a grid search in the area $\sigma_0 \in [0.05, 0.6] \times \sqrt{\nu} \in [0.05, 0.6]$, with an interval of 0.05 in each dimension. We evaluated the short-term forecast using the mean absolute error (MAE) averaged over all forecast weeks and all state locations (Fig. 4). Based on the overall performance for the 4 targets, we selected the combination of $\sigma_0 = 0.5$ and $\sqrt{\nu} = 0.2$. In state-level retrospective forecasting, we thus fixed the values $\sigma_0 = 0.5$ and $\sqrt{\nu} = 0.2$ and used the $\sigma^2_{y^o}$ form in Eq. (15).

Note 5 Uncertainty propagation

For each location i, we use a binary variable p_i to record whether it is included in the surveillance network ($p_i = 1$) or not ($p_i = 0$). The vector $\mathbf{p} = (p_1, \cdots, p_m)^T$ then fully represents the configuration of the surveillance network in a total of m locations. In the ensemble M/D/A forecasting system, the uncertainty of variables can be represented by their standard deviation, which can be estimated directly from the ensemble. We record the uncertainty of the infected and susceptible populations in a vector $\mathbf{x} = (\sigma_{I_1}, \cdots, \sigma_{I_m}, \sigma_{S_1}, \cdots, \sigma_{S_m})^T$ in a system with m locations. In the M/D/A framework, the uncertainty vector $\mathbf{x}$ undergoes two update processes: 1) $\mathbf{x}$ is reduced after assimilation of observations; and 2) the reduced $\mathbf{x}$ propagates per the metapopulation model detailed by Eqs. (1)-(2) (*41–43*). Next, we quantitatively examine the uncertainty propagation during each process.

Uncertainty reduction during data assimilation

Data assimilation can be formulated within the following state-space framework (*34*). Let $\mathbf{z}_t$ be the $n \times 1$ joint state vector at time t that contains unobserved variables, observed variables and parameters. The evolution of $\mathbf{z}_t$ follows a dynamical model

$$\frac{d\mathbf{z}_t}{dt} = F(\mathbf{z}_t, t). \tag{16}$$

The set of m observations $\mathbf{y}_t^o$ at time t is determined by

$$\mathbf{y}_t^o = \mathbf{H}\mathbf{z}_t + \boldsymbol{\varepsilon}_t, \tag{17}$$

where the observation operator $\mathbf{H}$ is an $m \times n$ matrix and $\boldsymbol{\varepsilon}_t$ is an $m \times 1$ observational error drawn from a distribution with mean 0 and covariance $\mathbf{R}_t$. Supposing that the ith observed variable locates at row $pos(i)$ of $\mathbf{z}_t$, $\mathbf{H}$ is defined by $\mathbf{H}(i, pos(i)) = 1$ for $i = 1, \cdots, m$ and 0 for all other elements. In our model, we have $\mathbf{z}_t = (I_1, S_1, y_1, \cdots, I_m, S_m, y_m, R_{0max}, R_{0min}, L, D, \theta)^T$ and $pos(i) = 3i$.

In state-space problems, the key objective is to obtain the posterior of the model state vector $\mathbf{z}_t$ given observations $\mathbf{y}^o_{1:t}$ before and at time t via Bayes rule: $p(\mathbf{z}_t|\mathbf{y}^o_{1:t}) \propto p(\mathbf{y}^o_t|\mathbf{z}_t)p(\mathbf{z}_t|\mathbf{y}^o_{1:t-1})$, where $p(\mathbf{z}_t|\mathbf{y}^o_{1:t-1})$ is the prior, $p(\mathbf{y}^o_t|\mathbf{z}_t)$ is the likelihood, and $p(\mathbf{z}_t|\mathbf{y}^o_{1:t})$ is the posterior. We use the EAKF method to update the prior to its posterior. In particular, Kalman filters assume a Gaussian prior, likelihood and posterior, each of which can be fully characterized by their respective means and covariances. Assume the mean and covariance of the prior are $\overline{\mathbf{z}}_{t,prior}$ and $\mathbf{\Sigma}_{t,prior}$. The posterior is a Gaussian distribution with variance (*34*)

$$\mathbf{\Sigma}_{t,post} = \left[(\mathbf{\Sigma}_{t,prior})^{-1} + \mathbf{H}^T\mathbf{R}_t^{-1}\mathbf{H}\right]^{-1} \tag{18}$$

and mean

$$\overline{\mathbf{z}}_{t,post} = \mathbf{\Sigma}_{t,post}\left[(\mathbf{\Sigma}_{t,prior})^{-1}\overline{\mathbf{z}}_{t,prior} + \mathbf{H}^T\mathbf{R}_t^{-1}\mathbf{y}^o_t\right]. \tag{19}$$

The uncertainty in the posterior can be measured by the variance $\mathbf{\Sigma}_{t,post}$ (or standard deviation) of the system state. In practice, to avoid computing the inverse of large matrices, the update is performed on pairs of state variables and observations (*34*). Specifically, for a single observation y^o_i from location i and a state variable z (e.g., the infected or susceptible population in a location), we can define an associated *two-dimensional* state vector $(y_i, z)^T$, the observation operator becomes $\mathbf{H} = (1, 0)$, the observational covariance $\mathbf{R}_t$ becomes a scalar $R_{t,i}$ (i.e., the OEV of the observation y^o_i), and the prior covariance becomes

$$\mathbf{\Sigma}_{t,prior} = \begin{pmatrix} \sigma^2_{y_i} & \sigma_{y_i z} \\ \sigma_{y_i z} & \sigma^2_z \end{pmatrix}, \tag{20}$$

where $\sigma^2_{y_i}$ and σ^2_z are the variances of y_i and z, and $\sigma_{y_i z}$ is the covariance between y_i and z. Using Eq. (18), the posterior variance of z becomes $\sigma^2_z\left\{1 - \sigma^2_{y_i z}/\left[\sigma^2_z(R_{t,i} + \sigma^2_{y_i})\right]\right\}$. In the EAKF update, we loop through all m observations, each of which is used to update all states in $\mathbf{z}_t$. For a specific state z, its variance after the EAKF update is

$$\sigma^2_{z,post} = \sigma^2_z \prod_{i=1}^{m}\left[1 - \frac{\sigma^2_{y_i z}}{\sigma^2_z(R_{t,i} + \sigma^2_{y_i})}\right]. \tag{21}$$

We can see that the impact of the ith observation on the variance of posterior z is dependent on the covariance $\sigma_{y_i z}$, its variance $R_{t,i}$, and the prior variance of the observed variable $\sigma^2_{y_i}$. If we quantify the uncertainty of z using standard deviation, the EAKF update will reduce σ_z to

$$\sigma_{z,post} = \sigma_z \sqrt{\prod_{i=1}^{m} \left[1 - \frac{\sigma^2_{y_i z}}{\sigma^2_z (R_{t,i} + \sigma^2_{y_i})} \right]}. \tag{22}$$

To verify Eq. (21), we ran the EAKF algorithm using the AFHSB data for 9 seasons. During each weekly data assimilation, we compared the actual reduction of σ^2_S and σ^2_I in the ensemble (denoted as $\Delta\sigma^2_S$ and $\Delta\sigma^2_I$), as well as the theoretical prediction using Eq. (21). Quantities used in Eq. (21) were calculated directly from the ensemble. The observed uncertainty reduction in the ensemble agrees well with the theoretical values (see Fig. 5).

Next we examine the impact of each observation y^o_i on the uncertainty vector $\mathbf{x}$. According to Eq. (22), assimilating the observation from location i would affect the uncertainty in all locations. We define $u^I_{j\leftarrow i}$ and $u^S_{j\leftarrow i}$ as the reduced fraction of variance for infected and susceptible populations in location j attributed to the observation from location i. Then from Eq. (22), we have

$$u^I_{j\leftarrow i} = \frac{\sigma^2_{y_i I_j}}{(R_{t,i} + \sigma^2_{y_i})\sigma^2_{I_j}}, u^S_{j\leftarrow i} = \frac{\sigma^2_{y_i S_j}}{(R_{t,i} + \sigma^2_{y_i})\sigma^2_{S_j}}. \tag{23}$$

Based on above analysis, the effect of data assimilation on uncertainty reduction can be encoded in a diagonal matrix $\mathbf{P} = diag(P_1, \cdots, P_m, P_{m+1}, \cdots, P_{2m})$, where

$$P_j = \sqrt{\prod_{i=1}^{m} (1 - p_i u^I_{j\leftarrow i})}, P_{j+m} = \sqrt{\prod_{i=1}^{m} (1 - p_i u^S_{j\leftarrow i})} \tag{24}$$

for $j = 1, \cdots, m$. In particular, the prior uncertainty vector $\mathbf{x}$ becomes $\mathbf{Px}$ after the EAKF update:

$$\mathbf{x} \Rightarrow \mathbf{Px}. \tag{25}$$

Note that $\mathbf{P}$ depends on both the surveillance system configuration $\mathbf{p}$ and time-varying factors

$u^I_{j\leftarrow i}$ and $u^S_{j\leftarrow i}$. In implementation, the quantities defining $u^I_{j\leftarrow i}$ and $u^S_{j\leftarrow i}$ can be computed numerically using the state-vector ensemble during data assimilation.

Uncertainty growth during model integration

For a sufficiently small uncertainty vector $\mathbf{x}$, its evolution after a certain time t in the metapopulation model can be approximated by a set of linear differential equations:

$$\frac{d\mathbf{x}}{dt} = \mathbf{J}(t)\mathbf{x}. \tag{26}$$

Here $\mathbf{J}(t)$ is the Jacobian matrix of the full nonlinear system of Eqs. (1)-(2) at time t. The growth of $\mathbf{x}$ in a short interval δt can be approximated by $\mathbf{x}(t+\delta t) \approx e^{\mathbf{J}(t)\delta t}\mathbf{x}(t)$.

After data assimilation, the uncertainty vector $\mathbf{Px}$ propagates per the metapopulation model for a short time interval δt:

$$\mathbf{Px} \Rightarrow \mathbf{MPx} \approx [\mathbf{I} + \mathbf{J}(t)\delta t]\mathbf{Px}, \tag{27}$$

where $\mathbf{I}$ is a $2m \times 2m$ unit matrix. Explicitly, the elements of matrix $\mathbf{M}$ are:

$$M_{ii} = 1 + \left(\frac{\beta_i S_i}{N_i} - \frac{1}{D} - \frac{\theta}{N_i}\sum_{j\neq i}\bar{C}^i_j\right)\delta t, \quad i = 1, \cdots, m, \tag{28}$$

$$M_{ij} = \theta\frac{\bar{C}^j_i}{N_j}\delta t, \quad i = 1, \cdots, m, j = 1, \cdots, m, i \neq j, \tag{29}$$

$$M_{ij} = \frac{\beta_i I_i}{N_i}\delta t, \quad i = 1, \cdots, m, j = i + m, \tag{30}$$

$$M_{ij} = \left(-\frac{1}{L} - \frac{\beta_i S_i}{N_i}\right)\delta t, \quad i = m+1, \cdots, 2m, j = i - m, \tag{31}$$

$$M_{ii} = 1 + \left(-\frac{1}{L} - \frac{\beta_i I_i}{N_i} - \frac{\theta}{N_i}\sum_{j\neq i}\bar{C}^i_j\right)\delta t, \quad i = m+1, \cdots, 2m, \tag{32}$$

$$M_{ij} = \theta\frac{\bar{C}^j_i}{N_j}\delta t, \quad i = m+1, \cdots, 2m, j = m+1, \cdots, 2m, i \neq j. \tag{33}$$

All other elements are zero. The state variables and parameters in $\mathbf{M}$ are set as the posterior ensemble mean values derived from the EAKF. The linear approximation was shown to be

valid for a few days for influenza transmission models. Typical respiratory disease surveillance releases data once per week; at this rate the linear approximation may become less accurate. As a consequence, we here limit our attention to short-term uncertainty propagation during $\delta t = 1$ day. Later retrospective forecast results indicate that this setting can improve near-term forecasts for ILI+ up to 4 weeks ahead. Other reasonable choices of δt yield similar optimization results.

Quantification of uncertainty propagation

Combining the above two processes, if the observation interval is sufficiently small, we can track the long-term uncertainty propagation in the "$\cdots \rightarrow$ data assimilation $\rightarrow$ model integration $\rightarrow$ data assimilation $\rightarrow \cdots$" cycle by successive application of Eq. (25) and Eq. (27). For influenza surveillance, operational data release frequency is typically one week, for which the linear approximation in Eq. (27) may become less accurate. As a consequence, in this work we limit our attention to the short-term evolution of the uncertainty vector $\mathbf{x}$ during a short time interval $\delta t = 1$ day. Specifically, we quantify the uncertainty growth through

$$\frac{\|\mathbf{MPx}\|^2}{\|\mathbf{x}\|^2} = \frac{\mathbf{x}^T\mathbf{P}^T\mathbf{M}^T\mathbf{MPx}}{\mathbf{x}^T\mathbf{x}}. \tag{34}$$

Here the norm of a vector $\mathbf{x}$ is given by $\|\mathbf{x}\|^2 = \mathbf{x}^T\mathbf{x}$. Equation (34) indicates that uncertainty will expand along the direction of the principal eigenvector of $\mathbf{L} \equiv \mathbf{P}^T\mathbf{M}^T\mathbf{MP}$ (*39,44–50*). The rate of expansion is determined by the dominant eigenvalue λ_1. The symmetry of $\mathbf{L}$ guarantees that λ_1 is a real number. Furthermore, numerical experiments suggest that λ_1 is always positive for influenza transmission dynamics. To suppress the growth of uncertainty, we here attempt to minimize λ_1.

Optimal surveillance problem

The above argument has transformed the task of selecting K optimal sentinels from m locations to a combinatorial optimization of the vector $\mathbf{p}$, with a constraint on the number of surveillance locations $\sum_{i=1}^{m} p_i = K$. The optimization objective is to minimize the dominant

eigenvalue λ_1 of the matrix $\mathbf{L}$. The selected locations are those with $p_i = 1$.

Because $\mathbf{L}$ is determined by time-varying parameters (e.g., $\beta_i(t)$) and state variables (e.g., $I_i(t)$ and $S_i(t)$), in order to find an optimal configuration of $\mathbf{p}$ that works for a range of prediction scenarios, we need to consider forecasts made at different times with different system states. Thus, instead of optimizing λ_1 at a given time for a specific system state, we should minimize the mean value of λ_1 averaged over different forecast times and possible system states. Mathematically, the optimal surveillance problem becomes

$$\mathbf{p}^* = \underset{\mathbf{p}\in\mathcal{P}}{\arg\min} \quad \langle\lambda_1(\mathbf{p}, t, \mathbf{z})\rangle \quad \text{subject to} \quad \sum_{i=1}^{m} p_i = K, p_i \in \{0, 1\}.$$

Here, $\lambda_1(\mathbf{p}, t, \mathbf{z})$ is the dominant eigenvalue of $\mathbf{L}$ at time t with system state $\mathbf{z}$, given the configuration of the surveillance network $\mathbf{p}$; $\mathcal{P}$ is the space of 2^m possible configurations of $\mathbf{p}$; K is the constraint on the number of surveillance locations; and $\mathbf{p}^*$ is the optimal configuration that minimizes the mean value of λ_1.

In order to calculate $\lambda_1(\mathbf{p}, t, \mathbf{z})$, we run weekly data assimilation in multiple seasons to estimate the system state $\mathbf{z}$ at each week. Using the surveillance network configuration $\mathbf{p}$ and the posterior model state $\mathbf{z}$ at time t, we obtain the matrices $\mathbf{P}$ and $\mathbf{M}$, and then compute the dominant eigenvalue λ_1 of $\mathbf{L}$ using the power method. The mean eigenvalue is averaged over $\lambda_1(\mathbf{p}, t, \mathbf{z})$ for different weeks and seasons. In particular, we used the matrices at each of 30 forecast weeks in each season, and the corresponding system states $\mathbf{z}$ were estimated using the posterior mean. At state level, we performed 10 independent realizations of the EAKF to infer the system state at each week. We solve this optimization problem using simulated annealing (*51*).

Note 6 Retrospective forecasting for influenza at the state level

System configurations To perform retrospective forecasting using the M/D/A system,

configurations such as the initial conditions of the ensemble and observation error variance (OEV, $\sigma^2_{y^o}$) need to be pre-assigned. At the state level, we used 300 ensemble members. Initial conditions were drawn using Latin Hypercube Sampling (LHS) from the following ranges (*52*): $S_i \in [0.5N_i, 0.85N_i]$, $I_i \in [0.0005N_i, 0.001N_i]$, $R_{0max} \in [2.0, 2.5]$, $R_{0min} \in [1.5, 2.0]$, $L \in [2, 10]$ years, $D \in [5, 7]$ days, $\theta \in [0, 4.5]$. The observed variable y in each location is initialized as 0 and is calculated during model integration. The configuration of OEV is reported in the last section.

Mapping influenza incidence rate to ILI+ rate

The metapopulation model describes influenza transmission in the general population, and the state variable I_i represents influenza prevalence at each location; however, the observation (i.e., the ILI+ rate) reflects the incidence of influenza type A among individuals visiting treatment facilities. To address this mismatch, during model integration we calculate influenza incidence as the fraction of *new* infections each week. In addition, during retrospective forecasting, this influenza incidence rate in the metapopulation model needs to be transformed to the observed ILI+ quantity (*7*). ILI+ measures the probability that a person seeking medical attention (event Med) is infected with influenza type A (event Flu): $p(Flu|Med)$. Denote $p(Flu)$ as the influenza incidence rate, i.e., the probability of infection with influenza type A in the general population, $p(Med)$ as the probability of visiting a healthcare facility for any reason, and $p(Med|Flu)$ as the probability of seeking treatment among individuals with influenza type A. Bayes rule yields

$$p(Flu) = p(Med)\frac{p(Flu|Med)}{p(Med|Flu)} = \gamma ILI+,$$

where $\gamma \equiv p(Med)/p(Med|Flu)$ is the ratio of the probability of visiting a doctor for any reason to the probability that persons with influenza type A seek medical care. The scaling parameter γ may be time-varying; however, without detailed real-time information, we assume

a constant γ for each location. In practice, we apply the scaling process during model integration and divide the weekly incidence rate by γ. With this scaling, the observed state variable y is mapped to ILI+, matching with observations in the AFHSB data.

We used model simulations to estimate the scaling parameter γ_i for each location i. Specifically, we ran a series of free simulations using the metapopulation model and obtained a distribution of the annual total influenza incidence rate for each location. We then compared the simulated annual incidence rate with the observed annual ILI+ rate (i.e., sum of weekly ILI+ rates) for each of the 35 states. We estimate γ_i as the ratio of the simulated rate of annual incidence averaged over 1,000 free simulations with random initialization to the average value of observed annual ILI+ (Fig. 6). The rescaled simulated distributions well capture the observed data, indicating that, under this scaling and parameter setting, the metapopulation model is capable of generating realistic variations in observed annual ILI+ rates. The scaling parameters for the 35 state locations are distributed around 2 (mean: 1.97, standard deviation: 0.58). During retrospective forecasting, we fixed these scaling parameters throughout all 9 seasons.

Performance of near-term predictions

We examined forecast accuracy for 4 short-term targets: 1- to 4-week ahead ILI+ rate. Surveillance networks with 1 to 35 states were used to generate retrospective forecasts. We compared the performance of the surveillance networks selected using the SA algorithm with those selected using three alternative heuristic methods – population size, number of commuters and population gradient. Population gradient, denoted by ∇Population, is defined as the ratio of population size at a given location to the average population size of its adjacent neighbors. For each alternate strategy, states with a larger population, total number of commuters (including both incoming and outgoing directions) or higher ∇Population were selected as surveillance networks. We evaluate the performance of forecast accuracy using two measures: mean absolute error (MAE) and log score (*53–55*). MAE is calculated as the difference between the predicted

ensemble mean and observed ILI+ rate. Log score is defined as the log value of the probability assigned to the interval of width 0.01 centered at the observed ILI+ rate (0.005 on each side). For forecasts that missed this interval, we used a floor value of -6, which is smaller than the minimal log score $\log(1/300)$ if at least one ensemble member falls within the interval.

We present the comparison of MAE and log score for 1- to 4-week ahead prediction in Fig. 7A. In general, SA outperforms Population, Commuter and ∇Population in most cases, consistent with the findings for 1-week ahead prediction. The advantage of SA decreases for longer forecast horizons. These results imply that, although the SA algorithm optimizes uncertainty propagation for a short time interval, its improvement lasts for predictions up to 4 weeks ahead.

In order to examine whether the SA algorithm statistically significantly outperforms the other three strategies, we performed a Wilcoxon signed-rank test on three pairs of methods: SA-Population, SA-Commuter, and SA-∇Population. The Wilcoxon signed-rank test is a non-parametric statistical test that compares two paired samples (here, paired MAEs or log scores generated by both examined methods for the same location at the same forecast week) to assess whether their population mean ranks differ (*56*). We performed a two-sided test to return a p-value indicating that SA outperforms the other method. We calculated the p-values for the three pairs of comparison (SA-Population, SA-Commuter, and SA-∇Population) for each of four targets (X1 to X4) (Fig. 7B). The p-values reported in the main text are the maximal p-values among all three tests (i.e., the worst case).

In Fig. 8 and Fig. 9, we compared the forecast error and log score at different times relative to the predicted lead (negative: before predicted peak week; positive: after predicted peak week). Retrospective forecasts in 35 states and 9 seasons were generated using surveillance networks consisting of 20 states. The SA algorithm outperforms other methods, particularly after the predicted peak week.

Note 7 Forecasting at county level

We validated the population gradient method at the county level using model-generated outbreaks for all 3,108 counties in the continental US. County-to-county commuting data were obtained from the US census survey (*5*), and daily absolute humidity data were derived from the North American Land Data Assimilation System at the centroid of each county (*4*).

We generated synthetic outbreaks using the metapopulation model in Eqs. (1)-(2). The initial conditions were drawn using LHS from the following ranges: $S_i \in [0.65N_i, 0.75N_i]$, $I_i \in [0.0005N_i, 0.001N_i]$, $R_{0max} \in [2.0, 2.5]$, $R_{0min} \in [1.5, 2.0]$, $L \in [2, 10]$ years, $D \in [5, 7]$ days, $\theta \in [0, 1]$. We started a 30-week simulation from the 273rd day of the year (typically in late September or early October) and recorded the weekly incidence rate in all counties. To mimic the data streams collected from sentinel surveillance, we added two sources of noise to the time series. First, we randomly generated a scaling parameter for each county, following a Gaussian distribution $\gamma \sim \mathcal{N}(2, 0.2^2)$. The weekly incidence rate in each location was divided by the corresponding scaling parameter γ to generate the ILI+ rate in healthcare facilities. Second, we imposed observational errors to the obtained ILI+ rates. The observational errors were also Gaussian distributed, with zero mean and OEV related to sampling. In the state-level data, we observed a correlation between the number of annual laboratory tests and population. We fitted a linear function at the log scale, and generated annual sample numbers at county level using the function: $\log(n_{sample}) = (0.4063 \log(pop) + 1.161) \times (1 + 0.2\varepsilon)$, where pop is population size and ε is a Gaussian noise $\varepsilon \sim \mathcal{N}(0, 1)$. Because more samples were collected around outbreak peaks, the annual sample number was then distributed to each week weighted by ILI+ to generate weekly sample numbers: $n_{sample}(t) = y_t n_{sample} / \sum_t y_t$, where y_t is the simulated ILI+ rate at week t. The OEV at week t was set as OEV$(t) = 10^{-8} + (\sum_{t'=t-2}^{t} y_{t'}/3)^2 / n_{sample}(t)$, a baseline plus a term determined by influenza activity from week $t-2$ to week t and weekly

sample number. For each synthetic outbreak, the scaling parameters and OEV were generated independently. To exclude unrealistic synthetic outbreaks, we used the following criterion to select initial conditions: 1) the largest peak intensity among all counties is smaller than 0.1; 2) at least 90% of counties have peak intensity larger than 0.005. We generated 20 synthetic outbreaks for validation.

The comparison of MAE and log score for 1- to 4-week ahead prediction is presented in Fig. 11A. Consistent with the findings for 1-week ahead prediction, ∇Population outperforms Population and Commuter in most cases. The horizontal bar on top of each panel shows the p-value of the Wilcoxon test that population gradient outperforms both Population and Commuter methods (Fig. 11B).

Note 8 Retrospective forecasting for HMPV and CoV

We performed retrospective forecasting for HMPV and CoV in 35 US states for the 2013-2014 to 2016-2017 seasons, using a forecast system similar to the one used for influenza. An SIRS metapopulation model was used to describe the transmission process, with variable and parameter ranges set as follows: $S_i \in [0.4N_i, 0.55N_i]$, $I_i \in [0.001N_i, 0.002N_i]$, $R_0 \in [2, 3.5]$, $L \in [2, 10]$ years, $D \in [2, 12]$ days, $\theta \in [0, 4.5]$. The scaling parameter and OEV in each state were determined using the same procedure as for influenza. In each season, 30 weekly forecasts were generated starting from the 40th week in each year. Surveillance networks consisting of 1 to 10 states designed using population gradient, Population and Commuters are compared. Observing more than 10 states provides nominal improvement, so we focus on a few key states. The forecast error and log score for 1- to 4-week ahead predictions are shown in Fig. 12. The results suggest two points: 1) For HMPV and CoV, real-time forecasting in locations without surveillance is possible; 2) the population gradient approach outperforms strategies based on population and number of commuters when selecting surveillance sites. Comparing HMPV

and CoV, the advantage of population gradient is more prominent for HMPV. This is possibly due to a relatively smaller number of laboratory tests for CoV, which leads to noisier CoV+ curves than HMPV+.

References

1. N. Marsden-Haug, V. B. Foster, P. L. Gould, E. Elbert, H. Wang, J. A. Pavlin, Code-based syndromic surveillance for influenzalike illness by International Classification of Diseases, Ninth Revision. *Emerg. Infect. Dis.* **13**, 207 (2007).

2. E. Goldstein, C. Viboud, V. Charu, M. Lipsitch, Improving the estimation of influenza-related mortality over a seasonal baseline. *Epidemiology* **23**, 829-838 (2012).

3. P. Riley, M. Ben-Nun, R. Armenta, J. A. Linker, A. A. Eick, J. L. Sanchez, D. George, D. P. Bacon, S. Riley, Multiple estimates of transmissibility for the 2009 influenza pandemic based on influenza-like-illness data from small US military populations. *PLOS Comput. Biol.* **9**, e1003064 (2013).

4. B. A. Cosgrove, D. Lohmann, K. E. Mitchell, P. R. Houser, E. F. Wood, J. C. Schaake, A. Robock, C. Marshall, J. Sheffield, Q. Duan, L. Luo, R. W. Higgins, R. T. Pinker, J. D. Tarpley, J. Meng, Real-time and retrospective forcing in the North American Land Data Assimilation System (NLDAS) project. *J. Geophys. Res.* **108**, 8842 (2003).

5. United States Census Bureau, County to county commuting data. www.census.gov/topics/employment/commuting.html Accessed Feb 18, 2019.

6. J. Shaman, A. Karspeck, Forecasting seasonal outbreaks of influenza. *Proc. Natl. Acad. Sci. U.S.A.* **109**, 20425-20430 (2012).

7. J. Shaman, A. Karspeck, W. Yang, J. Tamerius, M. Lipsitch, Real-time influenza forecasts during the 20122013 season. *Nat. Commun.* **4**, 2837 (2013).

8. S. Pei, J. Shaman, Counteracting structural errors in ensemble forecast of influenza outbreaks. *Nat. Commun.* **8**, 925 (2017).

9. D. Osthus, J. Gattiker, R. Priedhorsky, S. Y. Del Valle, Dynamic Bayesian influenza forecasting in the United States with hierarchical discrepancy. *Bayesian Anal.* 10.1214/18-BA1117 (2018).

10. S. Pei, S. Kandula, W. Yang, J. Shaman, Forecasting the spatial transmission of influenza in the United States. *Proc. Natl. Acad. Sci. U.S.A.* **115**, 2752-2757 (2018).

11. J. B. S. Ong, I. Mark, C. Chen, A. R. Cook, H. C. Lee, V. J. Lee, R. T. P. Lin, P. A. Tambyah, L. G. Goh, Real-time epidemic monitoring and forecasting of H1N1-2009 using influenza-like illness from general practice and family doctor clinics in Singapore. *PLOS ONE* **5**, e10036 (2010).

12. W. Yang, M. Lipsitch, J. Shaman, Inference of seasonal and pandemic influenza transmission dynamics. *Proc. Natl. Acad. Sci. U.S.A.* **112**, 2723-2738 (2015).

13. W. Yang, A. Karspeck, J. Shaman, Comparison of filtering methods for the modeling and retrospective forecasting of influenza epidemics. *PLOS Comput. Biol.* **10**, e1003583 (2014).

14. S. Kandula, T. Yamana, S. Pei, W. Yang, H. Morita, J. Shaman, Evaluation of mechanistic and statistical methods in forecasting influenza-like illness. *J. R. Soc. Interface* **15**, 20180174 (2018).

15. D. Osthus, K. S. Hickmann, P. C. Caragea, D. Higdon, S. Y. Del Valle, Forecasting seasonal influenza with a state-space SIR model. *Ann. Appl. Stat.* **11**, 202-224 (2017).

16. T. Yamana, S. Kandula, J. Shaman, Superensemble forecasts of dengue outbreaks. *J. R. Soc. Interface* **13**, 20160410 (2016).

17. J. Reis, J. Shaman, Retrospective parameter estimation and forecast of respiratory syncytial virus in the United States. *PLOS Comput. Biol.* **12**, e1005133 (2016).

18. N. B. DeFelice, E. Little, S. R. Campbell, J. Shaman, Ensemble forecast of human West Nile virus cases and mosquito infection rates. *Nat. Commum.* **8**, 14592 (2017).

19. W. Yang, W. Zhang, D. Kargbo, R. Yang, Y. Chen, Z. Chen, A. Kamara, B. Kargbo, S. Kandula, A. Karspeck, C. Liu, J. Shaman, Transmission network of the 2014-2015 Ebola epidemic in Sierra Leone. *J. R. Soc. Interface* **12**, 20150536 (2015).

20. S. Pei, F. Morone, F. Liljeros, H. Makse, J. L. Shaman, Inference and control of the nosocomial transmission of methicillin-resistant Staphylococcus aureus. *eLife* **7**, e40977 (2018).

21. J. Shaman, V. E. Pitzer, C. Viboud, B. T. Grenfell, M. Lipsitch, Absolute humidity and the seasonal onset of influenza in the continental United States. *PLOS Biol.* **8**, e1000316 (2010).

22. J. Shaman, M. Kohn, Absolute humidity modulates influenza survival, transmission, and seasonality. *Proc. Natl. Acad. Sci. U.S.A.* **106**, 3243-3248 (2009).

23. C. Viboud, O. N. Bjørnstad, D. L. Smith, L. Simonsen, M. A. Miller, B. T. Grenfell, Synchrony, waves, and spatial hierarchies in the spread of influenza. *Science* **312**, 447-451 (2006).

24. D. Brockmann, D. Helbing, The hidden geometry of complex, network-driven contagion phenomena. *Science* **342**,1337-1342 (2013).

25. J. R. Gog, S. Ballesteros, C. Viboud, L. Simonsen, O. N. Bjørnstad, J. Shaman, D. L. Chao, F. Khan, B. T. Grenfell, Spatial transmission of 2009 pandemic influenza in the US. *PLOS Comput. Biol.* **10**, e1003635 (2014).

26. V. Charu, S. Zeger, J. Gog, O. N. Bjørnstad, S. Kissler, L. Simonsen, B. T. Grenfell, C. Viboud, Human mobility and the spatial transmission of influenza in the United States. *PLOS Comput. Biol.* **13**, e1005382 (2017).

27. V. Belik, T. Geisel, D. Brockmann, Natural human mobility patterns and spatial spread of infectious diseases. *Phys. Rev. X* **1**, 011001 (2011).

28. M. J. Keeling, P. Rohani, Estimating spatial coupling in epidemiological systems: a mechanistic approach. *Ecol. Lett.* **5**, 20-29 (2002).

29. V. Colizza, A. Barrat, M. Barthélemy, A. Vespignani, The role of the airline transportation network in the prediction and predictability of global epidemics. *Proc. Natl. Acad. Sci. U.S.A.* **103**, 2015-2020 (2006).

30. S. Riley, Large-scale spatial-transmission models of infectious disease. *Science* **316**, 1298-1301 (2007).

31. D. Balcan, V. Colizza, B. Gonçalves, H. Hu, J. J. Ramasco, A. Vespignani, Multiscale mobility networks and the spatial spreading of infectious diseases. *Proc. Natl. Acad. Sci. U.S.A.* **106**, 21484-21489 (2009).

32. M. J. Keeling, L. Danon, M. C. Vernon, T. A. House, Individual identity and movement networks for disease metapopulations. *Proc. Natl. Acad. Sci. U.S.A.* **107**, 8866-8870 (2010).

33. W. Yang, D. R. Olson, J. Shaman, Forecasting influenza outbreaks in boroughs and neighborhoods of New York City. *PLOS Comput. Biol.* **12**, e1005201 (2016).

34. J. L. Anderson, An ensemble adjustment Kalman filter for data assimilation. *Mon. Weather Rev.* **129**, 2884-2903 (2001).

35. G. Evensen, Data Assimilation: The ensemble Kalman filter (Springer, New York, NY, 2008).

36. M. S. Arulampalam, S. Maskell, N. Gordon, T. Clapp, A tutorial on particle filters for online nonlinear/non-Gaussian Bayesian tracking. *IEEE Trans. Signal Process* **50**, 174-188 (2002).

37. C. Andrieu, A. Doucet, R. Holenstein, Particle Markov chain Monte Carlo methods. *J. R. Stat. Soc. B* **72**, 269-342 (2010).

38. E. L. Ionides, C. Bretó, A. A. King, Inference for nonlinear dynamical systems. *Proc. Natl. Acad. Sci. U.S.A.* **103**, 18438-18443 (2006).

39. S. Pei, M. A. Cane, J. Shaman, Predictability in process-based ensemble forecast of influenza. *PLOS Comput. Biol.* **15**, e1006783 (2019).

40. A. Seijas-Macías, A. Oliveira, An approach to distribution of the product of two normal variables. *Discussiones Mathematicae Probability and Statistics* **32**, 87-99 (2012).

41. A. Carrassi, A. Trevisan, F. Uboldi, Adaptive observations and assimilation in the unstable subspace by breeding on the data-assimilation system. *Tellus A* **59**, 101-113 (2007).

42. A. Trevisan, F. Uboldi, Assimilation of standard and targeted observations within the unstable subspace of the observation-analysis-forecast cycle system. *J. Atmos. Sci.* **61**, 103-113 (2004).

43. F. Uboldi, A. Trevisan, Detecting unstable structures and controlling error growth by assimilation of standard and adaptive observations in a primitive equation ocean model. *Nonlinear Process. Geophys.* **13**, 67-81 (2006).

44. C. Nicolis, Dynamics of model error: Some generic features. *J. Atmos. Sci.* **60**, 2208-2218 (2003).

45. T. N. Palmer, G. J. Shutts, R. Hagedorn, F. J. Doblas-Reyes, T. Jung, M. Leutbecher, Representing model uncertainty in weather and climate prediction. *Annu. Rev. Earth Planet. Sci.* **33**, 163-193 (2005).

46. R. Buizza, T. N. Palmer, The singular-vector structure of the atmospheric global circulation. *J. Atmos. Sci.* **52**, 1434-1456 (1995).

47. T. N. Palmer, R. Gelaro, J. Barkmeijer, R. Buizza, Singular vectors, metrics, and adaptive observations. *J. Atmos. Sci.* **55**, 633-653 (1998).

48. R. Buizza, J. Tribbia, F. Molteni, T. Palmer, Computation of optimal unstable structures for a numerical weather prediction model. *Tellus A* **45**, 388-407 (1993).

49. T. M. Hamill, C. Snyder, R. E. Morss, A comparison of probabilistic forecasts from bred, singular-vector, and perturbed observation ensembles. *Mon. Weather Rev.* **128**, 1835-1851 (2000).

50. T. N. Palmer, Predicting uncertainty in forecasts of weather and climate. *Rep. Prog. Phys.* **63**, 71-116 (2002).

51. S. Kirkpatrick, C. D. Gelatt, M. P. Vecchi, Optimization by simulated annealing. *Science* **220**, 671-680 (1983).

52. B. Tang, Orthogonal array-based Latin hypercubes. *J. Am. Stat. Assoc.* **88**, 1392-1397 (1993).

53. M. Biggerstaff, D. Alper, M. Dredze, S. Fox, I. C. Fung, K. S. Hickmann, B. Lewis, R. Rosenfeld, J. Shaman, M. H. Tsou, P. Velardi, A. Vespignani, L. Finelli, the Influenza Forecasting Contest Working Group, Results from the centers for disease control and preventions predict the 2013-2014 Influenza Season Challenge. *BMC Infect. Dis.* **16**, 357 (2016).

54. M. Biggerstaff, M. Johansson, D. Alper, L. C. Brooks, P. Chakraborty, D. C. Farrow, S. Hyun, S. Kandula, C. McGowan, N. Ramakrishnan, R. Rosenfeld, J. Shaman, R. Tibshirani, R. J. Tibshirani, A. Vespignani, W. Yang, Q. Zhang, C. Reed, Results from the second year of a collaborative effort to forecast influenza seasons in the United States. *Epidemics* **24**, 26-33 (2018).

55. C. J. McGowan, M. Biggerstaff, M. Johansson, K. M. Apfeldorf, M. Ben-Nun, L. Brooks, M. Convertino, M. Erraguntla, D. C. Farrow, J. Freeze, S. Ghosh, S. Hyun, S. Kandula, J. Lega, Y. Liu, N. Michaud, H. Morita, J. Niemi, N. Ramakrishanan, E. L. Ray, N. G. Reich, P. Riley, J. Shaman, R. Tibshirani, A. Vespiganani, Q. Zhang, C. Reed, the Influenza Forecasting Working Group, Collaborative efforts to forecast seasonal influenza in the United States, 2015-2016. *Sci. Rep.* **9**, 683 (2019).

56. F. Wilcoxon, Individual comparisons by ranking methods. *Biometrics Bulletin* **1**, 80-83 (1945).

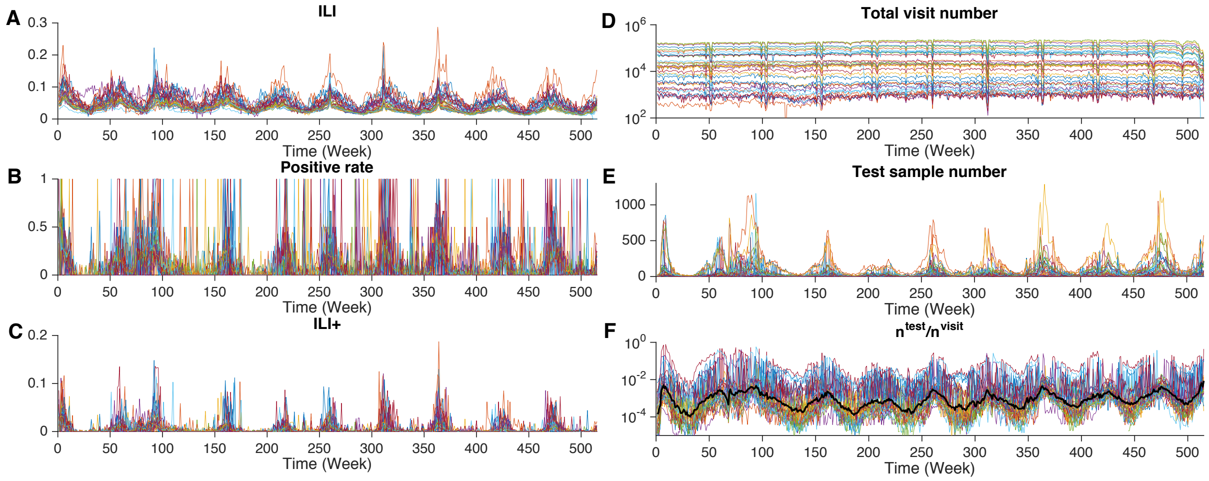

Figure 1: **The influenza surveillance data.** The weekly ILI rate (A), influenza type A positivity rate (B), ILI+ rate (C), the weekly number of total visits to MTFs (D), number of samples sent for laboratory testing (E) and the ratio of total visit number to tested sample number (F) in 35 states in the AFHSB dataset. All time series start from the first week of 2008 and last for 520 weeks.

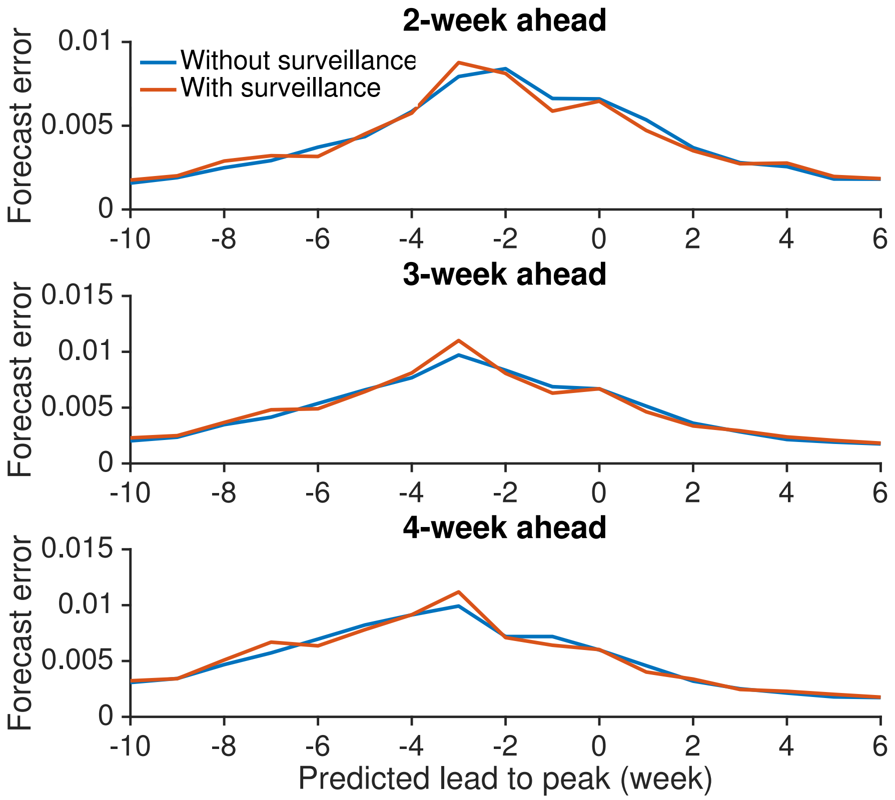

Figure 2: **Near-term predictions for influenza outbreaks with incomplete data.** Comparison of forecast MAE for 2- to 4-week ahead predictions with and without surveillance data. A set of forecasts was generated with data inputs from all 35 state locations over 9 seasons, and a second set of 35 forecasts over 9 seasons was generated with data inputs from 34 state locations, omitting data from one state location in turn. The forecast MAE in the examined state at each predicted lead was averaged over all 35 locations for versions with (orange) and without (blue) surveillance data.

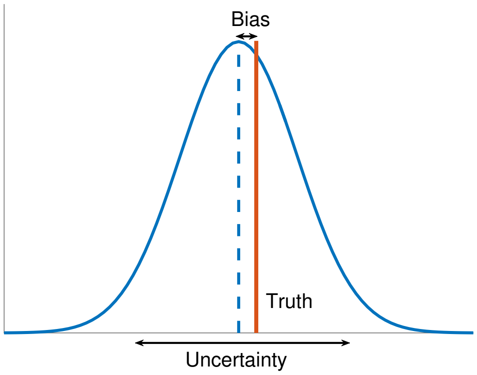

Figure 3: **Illustration of estimation bias and uncertainty.** The distribution shows the posterior estimation of a state variable. The vertical red line indicates the true value. The distance from the estimation mean (vertical dashed blue line) to the truth is defined as the estimation bias. The standard deviation of the posterior is defined as the estimation uncertainty. When the inference is not divergent from the truth, the estimation bias is smaller than or of the same order of magnitude as the estimation uncertainty.

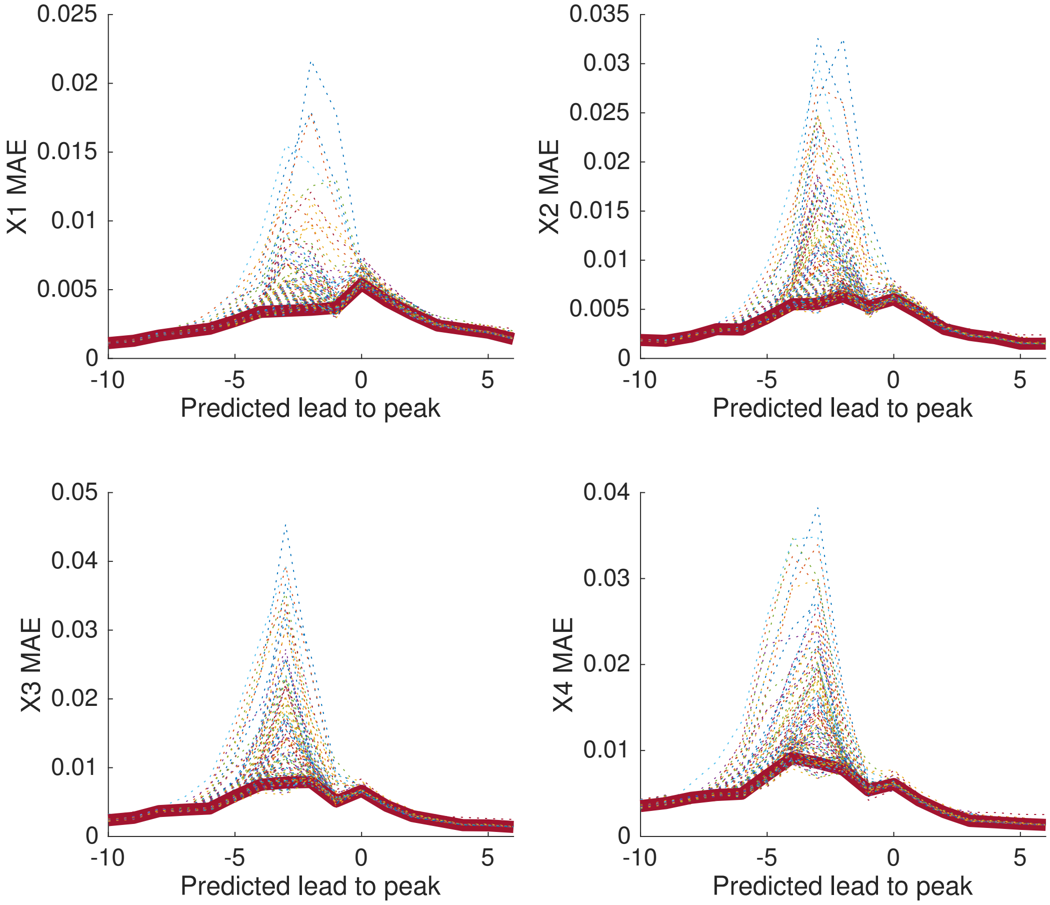

Figure 4: **Optimizing OEV for retrospective forecasting of influenza outbreaks.** The forecast MAE for 1- to 4-week ahead predictions grouped by lead week to predicted peak. A negative predicted lead indicates the peak is predicted to occur in the future, and a positive lead indicates the peak is predicted to have already passed. The curves corresponding to the selected parameter combination ($\sigma_0 = 0.5, \sqrt{\nu} = 0.2$) are highlighted by thickened lines. Curves for other combinations are shown as dotted lines.

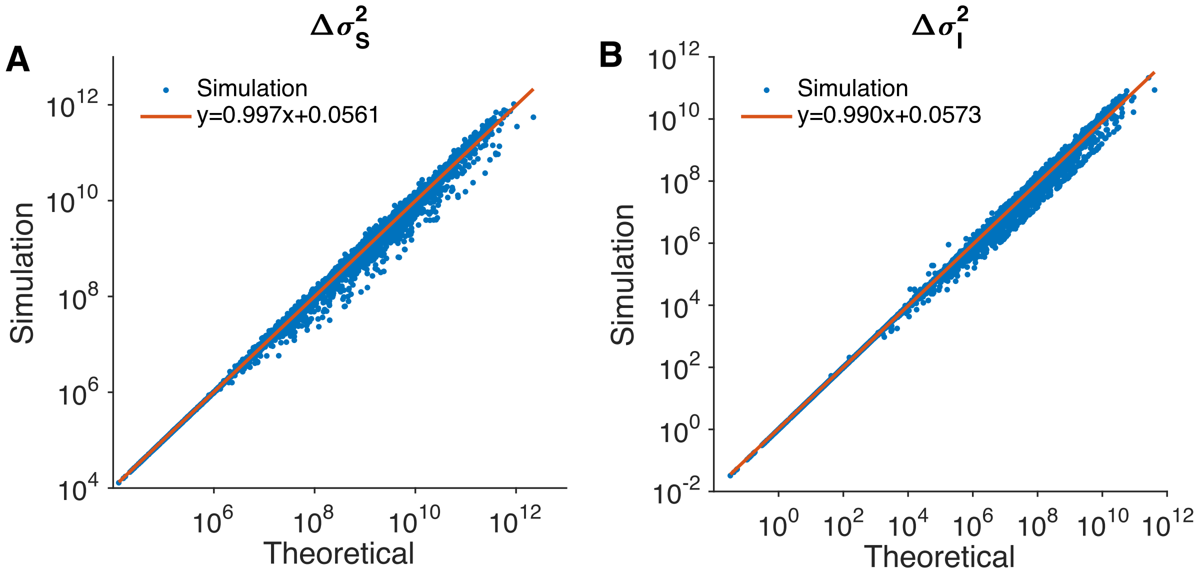

Figure 5: **Validation of the theoretical analysis of uncertainty reduction.** The theoretical prediction of the reduction in σ_S^2 (A) and σ_I^2 (B) (x-axis) agrees well with the values obtained directly from the ensemble in the EAKF (y-axis). The red lines are linear fittings. Each dot represents the result at each week per state.

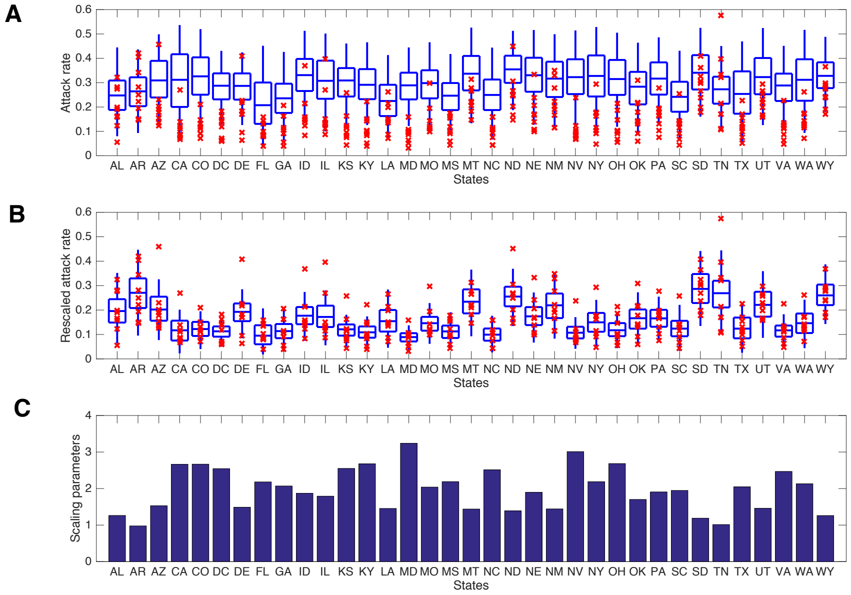

Figure 6: **Scaling of ILI+ rates.** (A) Distributions of attack rate in 35 states obtained from 1,000 model simulations (blue boxes: interquartile, whiskers: 95% CI) and the observed annual total ILI+ rate in 9 seasons (red crosses). (B) Most observed attack rates lie within the interquartile of the rescaled attack rate distributions. (C) Scaling parameters for 35 states. Analyses were performed for 35 states and the distributions were obtained from $n = 1,000$ independent experiments.

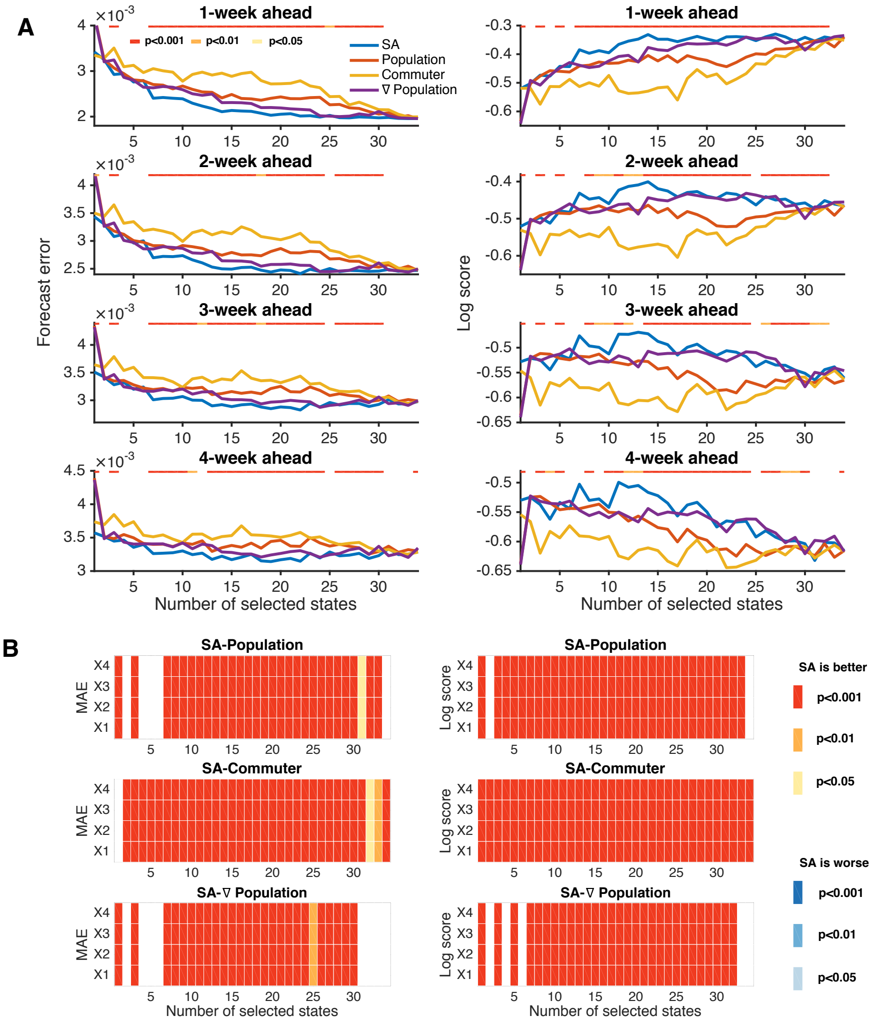

Figure 7: **Performance for 1- to 4-week ahead influenza prediction in 35 US states.** (A) We compare forecast error and log score for 1- to 4-week ahead ILI+ prediction in retrospective forecasts for 35 states and 9 seasons using surveillance networks selected by different methods. The horizontal bar on top of each panel shows the p-value for the case that SA outperforms the other three methods (two-sided Wilcoxon signed-rank test; red: $p < 0.001$, orange: $p < 0.01$, none: $p \geq 0.01$). (B) Results from the two-sided Wilcoxon signed-rank test at the state level. Tests for three combinations, i.e., SA-Population, SA-Commuter and SA-∇Population, are presented. We use colors to indicate 1) whether SA is better than the competing method, and use hues to indicate 2) the p-value of the test. Tests were performed for four targets (X1 to X4) using two measures (MAE and log score).

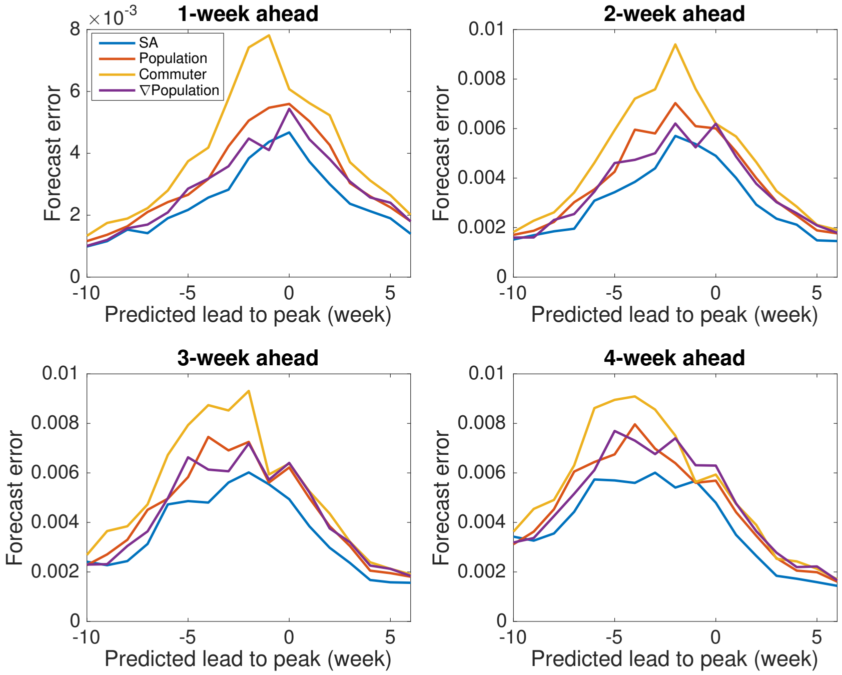

Figure 8: **Forecast error for 1- to 4-week ahead influenza prediction in 35 US states using data from 20 states.** We compare forecast error for 1- to 4-week ahead ILI+ prediction in retrospective forecasts for 35 states and 9 seasons using surveillance networks consisting of 20 states selected by different methods. The forecast error at each predicted lead (negative/positive: before/after predicted peak) is presented.

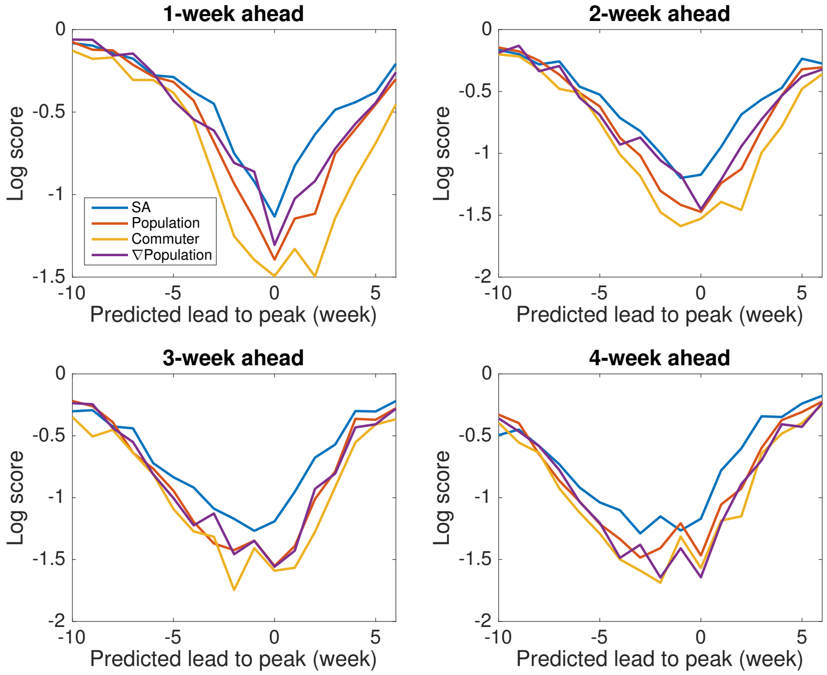

Figure 9: **Log score for 1- to 4-week ahead influenza prediction in 35 US states using data from 20 states.** We compare log score for 1- to 4-week ahead ILI+ prediction in retrospective forecasts for 35 states and 9 seasons using surveillance networks consisting of 20 states selected by different methods. The log score at each predicted lead (negative/positive: before/after predicted peak) is presented.

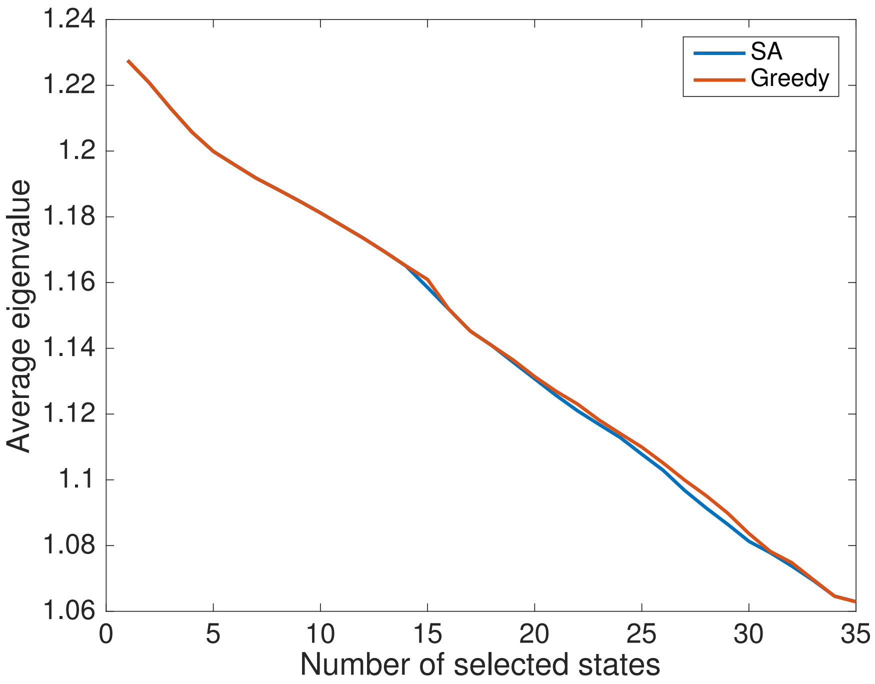

Figure 10: **The average eigenvalues of surveillance networks selected by SA and the greedy algorithm.** In the greedy algorithm, we sequentially select locations that lead to the largest marginal reduction of the average eigenvalue.

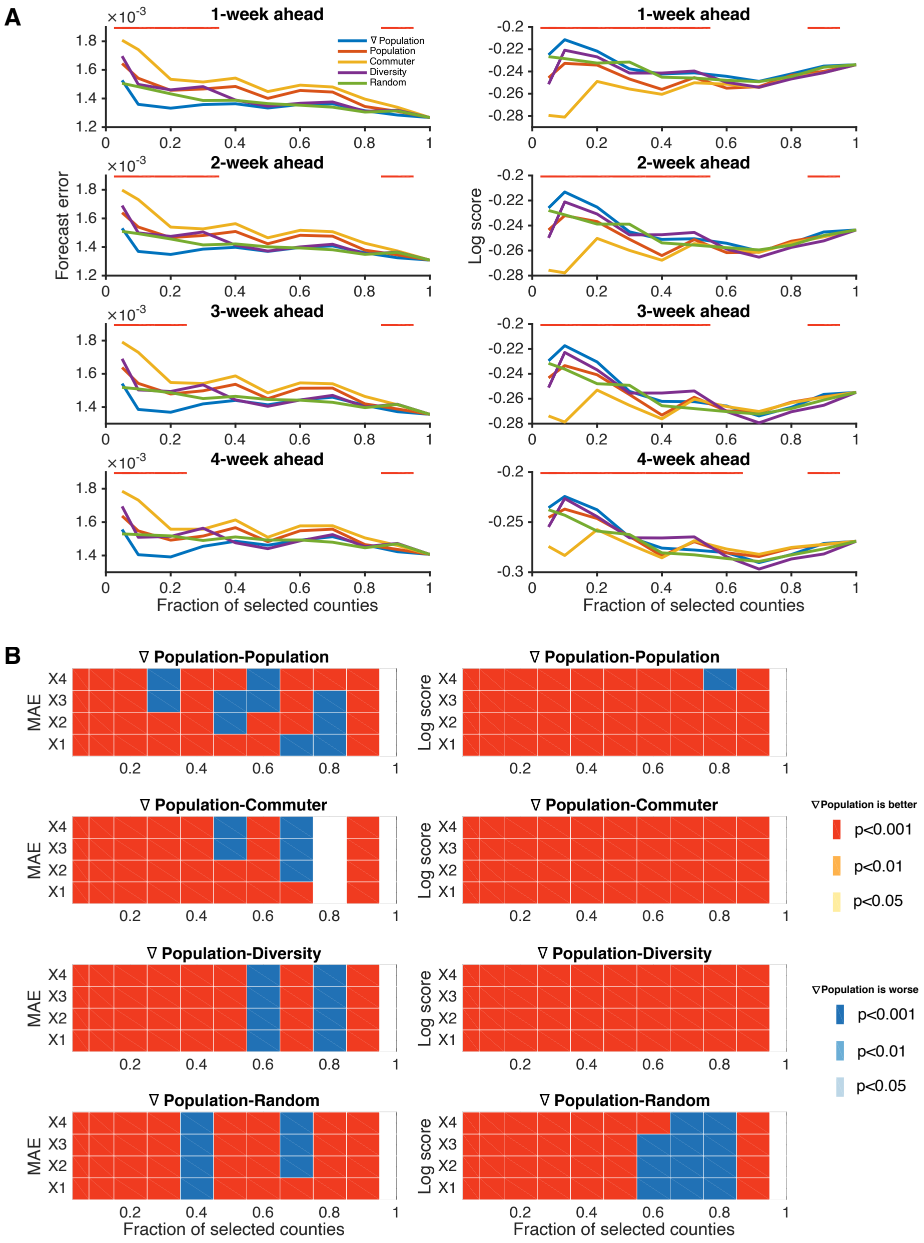

Figure 11: **Forecasting at county level for synthetic influenza outbreaks.** We compare forecast error and log score for 1- to 4-week ahead influenza predictions in county-level forecast for 20 synthetic outbreaks, using surveillance networks selected by different methods. Surveillance networks consist of 5%, 10%, 20% up to 100% of all counties. The horizontal bar on top of each panel shows the p-value for the case that ∇Population outperforms the other methods (two-sided Wilcoxon signed-rank test; red: $p < 0.001$, orange: $p < 0.01$, none: $p \geq 0.01$).

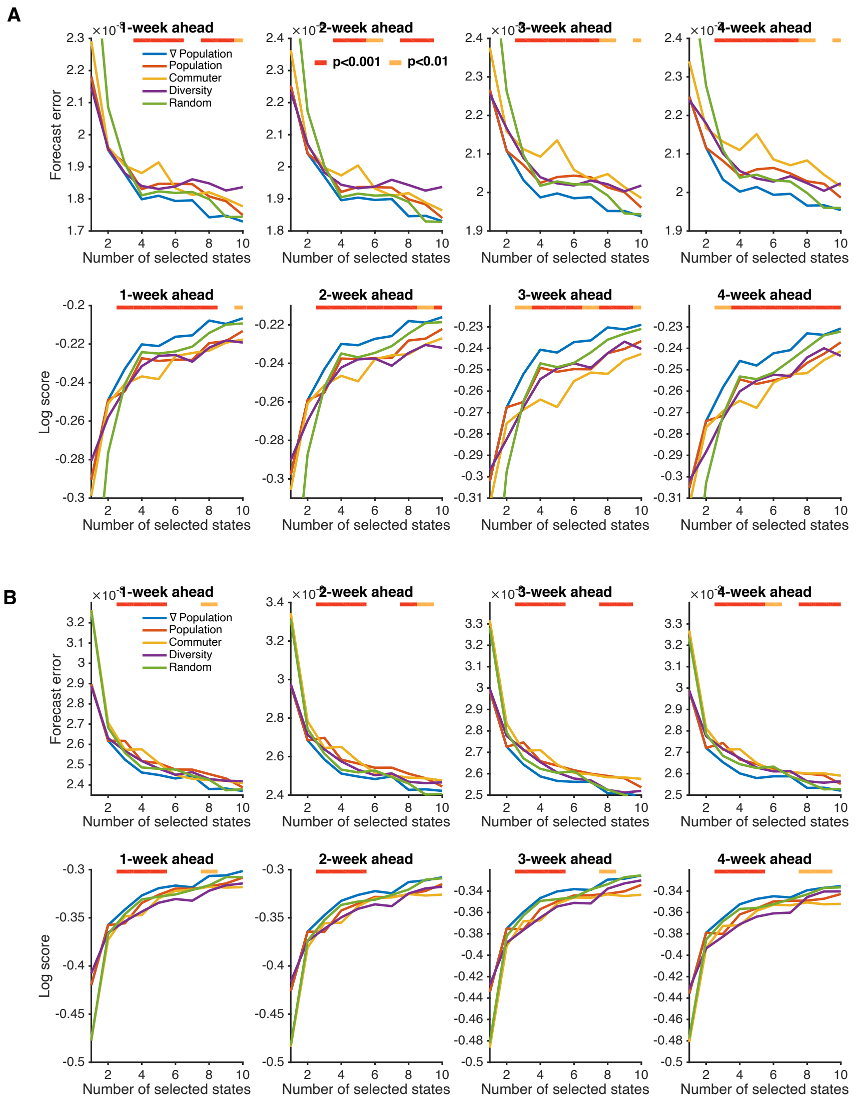

Figure 12: **Performance for 1- to 4-week ahead prediction for HMPV+ and CoV+ in 35 US states.** (A) We compare forecast error and log score for 1- to 4-week ahead HMPV+ prediction in retrospective forecasts for 35 states and 4 seasons using surveillance networks selected by different methods. The horizontal bar on top of each panel shows the p-value for the case that population gradient outperforms the other methods (two-sided Wilcoxon signed-rank test; red: $p < 0.001$, orange: $p < 0.01$, none: $p \geq 0.01$). (B) Same results for CoV+.
