# Supplementary material for: Optimizing respiratory virus surveillance networks using uncertainty propagation
Source: Nat Commun. 2021 Jan 11;12:222. doi: 10.1038/s41467-020-20399-3 (PMC7801666; doi:10.1038/s41467-020-20399-3)
Supplement: Supplementary file 3 — Reporting Summary [file 41467_2020_20399_MOESM3_ESM.pdf]

nature research

Corresponding author(s): Sen Pei

Last updated by author(s): 2020/11/23

Reporting Summary

Nature Research wishes to improve the reproducibility of the work that we publish. This form provides structure for consistency and transparency in reporting. For further information on Nature Research policies, see Authors & Referees and the Editorial Policy Checklist .

Statistics

For all statistical analyses, confirm that the following items are present in the figure legend, table legend, main text, or Methods section.

n/a	Confirmed	
☐	☒	The exact sample size (*n*) for each experimental group/condition, given as a discrete number and unit of measurement
☒	☐	A statement on whether measurements were taken from distinct samples or whether the same sample was measured repeatedly
☐	☒	The statistical test(s) used AND whether they are one- or two-sided<br>*Only common tests should be described solely by name; describe more complex techniques in the Methods section.*
☒	☐	A description of all covariates tested
☒	☐	A description of any assumptions or corrections, such as tests of normality and adjustment for multiple comparisons
☐	☒	A full description of the statistical parameters including central tendency (e.g. means) or other basic estimates (e.g. regression coefficient) AND variation (e.g. standard deviation) or associated estimates of uncertainty (e.g. confidence intervals)
☐	☒	For null hypothesis testing, the test statistic (e.g. *F*, *t*, *r*) with confidence intervals, effect sizes, degrees of freedom and *P* value noted<br>*Give P values as exact values whenever suitable.*
☐	☒	For Bayesian analysis, information on the choice of priors and Markov chain Monte Carlo settings
☒	☐	For hierarchical and complex designs, identification of the appropriate level for tests and full reporting of outcomes
☒	☐	Estimates of effect sizes (e.g. Cohen's *d*, Pearson's *r*), indicating how they were calculated

Our web collection on statistics for biologists contains articles on many of the points above.

Software and code

Policy information about availability of computer code

Data collection	Data are stored in MySql 8.0.
Data analysis	Data are analyzed using MATLAB R2015b. The EAKF algorithm is implemented in MATLAB R2015b. Custom codes developed for this study are available at https://github.com/SenPei-CU/SurveillanceOptimization.

For manuscripts utilizing custom algorithms or software that are central to the research but not yet described in published literature, software must be made available to editors/reviewers. We strongly encourage code deposition in a community repository (e.g. GitHub). See the Nature Research guidelines for submitting code & software for further information.

Data

Policy information about availability of data

All manuscripts must include a data availability statement. This statement should provide the following information, where applicable:

- Accession codes, unique identifiers, or web links for publicly available datasets
- A list of figures that have associated raw data
- A description of any restrictions on data availability

The US commuting data are available at https://www2.census.gov/programs-surveys/demo/tables/metro-micro/2015/commuting-flows-2015/table1.xlsx. The disease surveillance data that support the findings of this study are available from AFHSB but availability restrictions apply for these data, which were used under license for the current study and are not publicly available. Data are however available from the authors upon reasonable request and with permission of AFHSB.

Field-specific reporting

Please select the one below that is the best fit for your research. If you are not sure, read the appropriate sections before making your selection.

Ecological, evolutionary & environmental sciences study design

All studies must disclose on these points even when the disclosure is negative.

Study description	We compiled patient syndromic and laboratory test data from the US Department of Defense (DoD) Armed Forces Health Surveillance Branch (AFHSB) to estimate state-level disease activities of influenza, human metapneumovirus (HMPV) and coronavirus (CoV) in 35 US states.
Research sample	A total of 81,461,411 patient-line records of Influenza-like illness (ILI) related visits to permanent military treatment facilities (MTFs), each with a diagnosis containing at least one of the 29 International Classification of Diseases (ICD-9) codes associated with ILI, were collected. The ILI records span a period of nearly 18 years, from January 1st, 2000 to November 28th, 2017, and include approximately 1,000 MTFs located in 42 US states. Patients include both military personnel and other beneficiaries (e.g., spouses and children). In addition, results of laboratory tests for influenza performed on 845,081 samples during October 1st, 2006 to January 8th, 2018 were used. For HMPV and CoV, 44,887 and 27,435 laboratory test results from January 1st, 2013 to November 28th, 2017 were collected, respectively. Excluding seasons without laboratory tests and states with total ILI records fewer than 70,000 cases, we used data from 35 US states during 2008 to 2017 for influenza, and 2013 to 2017 for HMPV and CoV.
Sampling strategy	ILI-related medical visits were selected by 29 ICD-9 codes .
Data collection	Data were collected by AFHSB.
Timing and spatial scale	Influenza data were collected from 2008 to 2017 in 35 US states. HMPV and CoV data were collected from 2013 to 2017 in 35 US states. We aggregated the data as weekly records at the state level. For disease surveillance, weekly reporting at the state level is routinely used by the US CDC.
Data exclusions	No data were excluded from the analyses.
Reproducibility	Codes are publicly available at https://github.com/SenPei-CU/SurveillanceOptimization.
Randomization	Not applicable. We used population level healthcare data and did not define different groups.
Blinding	Not applicable. We used population level healthcare data and blinding is not needed.

Did the study involve field work? ☐ Yes ☒ No

Reporting for specific materials, systems and methods

We require information from authors about some types of materials, experimental systems and methods used in many studies. Here, indicate whether each material, system or method listed is relevant to your study. If you are not sure if a list item applies to your research, read the appropriate section before selecting a response.

Materials & experimental systems

n/a	Involved in the study
☒	☐ Antibodies
☒	☐ Eukaryotic cell lines
☒	☐ Palaeontology
☒	☐ Animals and other organisms
☐	☒ Human research participants
☒	☐ Clinical data

Methods

n/a	Involved in the study
☒	☐ ChIP-seq
☒	☐ Flow cytometry
☒	☐ MRI-based neuroimaging

Human research participants

Policy information about studies involving human research participants

Population characteristics	The population in the study includes both military personnel and other beneficiaries (e.g., spouses and children) who visited permanent military treatment facilities due to influenza-like illness, identified by ICD-9 codes. All age groups and genders are included.
Recruitment	Patients were selected from this passive surveillance system for influenza-like illness and identified by 29 ICD-9 codes.
Ethics oversight	Use of the deidentified dataset in this study was approved by AFHSB.

Note that full information on the approval of the study protocol must also be provided in the manuscript.
